# Histamine-related genes participate in the establishment of an immunosuppressive microenvironment and impact the immunotherapy response in hepatocellular carcinoma

**DOI:** 10.1007/s10238-024-01399-9

**Published:** 2024-06-17

**Authors:** Xianzhou Zhang, Peng Zheng, Bo Meng, Hao Zhuang, Bing Lu, Jun Yao, Feng Han, Suxia Luo

**Affiliations:** Department of Hepatic Biliary Pancreatic Surgery, The Affiliated Cancer Hospital of Zhengzhou University and Henan Cancer Hospital, Zhengzhou, 450008 China

**Keywords:** Hepatocellular carcinoma, Immunotherapy, Histamine, TME, TCR

## Abstract

Chronic inflammation is pivotal in the pathogenesis of hepatocellular carcinoma (HCC). Histamine is a biologically active substance that amplifies the inflammatory and immune response and serves as a neurotransmitter. However, knowledge of histamine’s role in HCC and its effects on immunotherapy remains lacking. We focused on histamine-related genes to investigate their potential role in HCC. The RNA-seq data and clinical information regarding HCC were obtained from The Cancer Genome Atlas (TCGA). After identifying the differentially expressed genes, we constructed a signature using the univariate Cox proportional hazard regression and least absolute shrinkage and selection operator (LASSO) analyses. The signature’s predictive performance was evaluated using a receiver operating characteristic curve (ROC) analysis. Furthermore, drug sensitivity, immunotherapy effects, and enrichment analyses were conducted. Histamine-related gene expression in HCC was confirmed using quantitative real-time polymerase chain reaction (qRT-PCR). A histamine-related gene prognostic signature (HRGPS) was developed in TCGA. Time-dependent ROC and Kaplan–Meier survival analyses demonstrated the signature’s strong predictive power. Importantly, patients in high-risk groups exhibited a higher frequency of *TP53* mutations, elevated immune checkpoint-related gene expression, and increased infiltration of immunosuppressive cells—indicating a potentially favorable response to immunotherapy. In addition, drug sensitivity analysis revealed that the signature could effectively predict chemotherapy efficacy and sensitivity. qRT-PCR results validated histamine-related gene overexpression in HCC. Our findings demonstrate that inhibiting histamine-related genes and signaling pathways can impact the therapeutic effect of anti-PD-1/PD-L1. The precise predictive ability of our signature in determining the response to different therapeutic options highlights its potential clinical significance.

**Supplementary Information:**

The online version contains supplementary material available at 10.1007/s10238-024-01399-9.

## Introduction

Hepatocellular carcinoma (HCC) is a widespread form of cancer and ranks as the third leading cause of cancer-related deaths worldwide. It constitutes about 90% of all primary liver cancer cases [1]. Early-stage HCC can be effectively treated using surgical interventions or ablation techniques. However, as the disease advances, current treatments, such as transarterial chemoembolization and systemic therapy, only offer palliative relief [2]. Due to an increasing understanding of the tumor immune microenvironment (TME) and immune escape pathways [3], immune checkpoint inhibitors (ICIs) have rapidly been developed in tumor therapy and demonstrated promising results in many solid tumors, including advanced HCC, non-small cell lung cancer, and metastatic melanoma [4–7]. In HCC, combination therapies based on ICIs continue to make significant breakthroughs in treatment. Several phase III clinical trials have investigated ICIs combined with antiangiogenic targeted drugs (AATDs) for advanced unresectable primary HCC, which have shown significantly improved clinical outcomes for patients with long-lasting benefits [8–10]. Although ICI-based combination regimens have become standard for advanced HCC, challenges like limited immunogenicity, insufficient or depleted immune cell infiltration, or a suppressive TME result in low response rates, benefiting only 30% of patients [11–13]. Therefore, effective markers that can predict immunotherapy efficacy are urgently needed. This will aid in screening potential targets while reducing medical costs.

HCC primarily arises due to chronic hepatitis and is infiltrated by multiple immune cells. TME of HCC can be categorized into inflammatory and noninflammatory types [14]. Histamine, an important inflammatory mediator derived from mast and tumor cells, is closely associated with allergic, inflammatory, and immune responses. Histamine receptors are G protein-coupled receptors, which can be divided into four isoforms: H1R, H2R, H3R, and H4R. H3R is primarily seen in neuronal cells, some tumor cells, and eosinophils. Immune cells majorly express H1R, H2R, and H4R. Histamine binds to different receptors mediating various immunomodulatory functions, which occasionally have contrasting effects [15]. The interaction between histamine and H1R on the surface of CD4T cells improves antigen presentation and Th1 cell differentiation, while the interaction between histamine and H2R on the surface of T cells stimulates the release of immunosuppressive factors like IL-10 and TGF-β [16–18]. In addition, histamine interacts with H1Rs on endothelial cell surface and promotes angiogenesis and TME remodeling [19]. Previous studies have focused on histamine upregulation in tumor tissues, leading to pro-tumor effects. However, the relationship between histamine levels in tumors and immune cell infiltration, as well as the efficacy of immunotherapy in HCC, has not been thoroughly investigated.

## Materials and methods

### Public data collection and preprocessing

The Cancer Genome Atlas-Liver Hepatocellular Carcinoma (TCGA-LIHC) datasets and clinical data of 365 HCC samples and 59 normal samples were obtained from TGCA. To validate our conclusions, bulk RNA-sequencing (RNA-seq) data of tumor tissues from 240 patients with HCC and their corresponding clinical information were downloaded from the International Cancer Genome Consortium database (https://dcc.icgc.org/) as a validation set. Additionally, bulk RNA-seq data of tumor tissues from 198 patients with HCC and their corresponding clinical information were downloaded from the Gene Expression Omnibus (GEO) database (GSE14520) for validation. The study obtained and compiled 1541 genes related to histamine from histamine-related pathways in Kyoto Encyclopedia of Genes and Genomes (KEGG), histamine-related genes in Hallmark, and specific genes linked to histamine. The IMvigor210 dataset includes bulk RNA-seq data and clinical characteristics of the patients treated with immunotherapy. Figure S1 presents the complete data analysis. A previous study [20] provided stem cell indices based on the transcriptome of normal liver and HCC tissues, referred to as mRNAsi in the following sections.

### Differentially expressed gene identification

Using the “limma” R package, 143 genes were compared between HCC and normal samples in the TCGA-LIHC cohort. Heatmaps and volcano maps were utilized to visualize the expression of histamine-related genes. The thresholds for differentially expressed genes (DEGs) were set as follows: the fold change (FC) for differential mRNA expression was |log2 fold change|≥ 1 and false discovery rate (FDR) < 0.05.

### Creation of the prognostic signature

Histamine-related DEGs were evaluated using a univariate Cox proportional hazard regression and a least absolute shrinkage and selection operator (LASSO) analyses (using “glmnet” R package) to avoid overfitting. Multivariate Cox proportional hazard regression was employed to develop the prognostic signature. Risk score = signature gene expression × matching coefficient for each patient. Patients were then stratified into high-risk or low-risk groups based on their median risk score.

### Validation of prognostic signature's predictive value

We calculated the risk score for each patient with HCC in the validation set using the same formula. The signature's predictive ability was assessed using Kaplan–Meier (KM) survival and ROC curves (employing R packages “survival” and “survminer”). The participants in the validation set were categorized using the same methodology as in the training set. Time-dependent ROC curves were utilized to evaluate the efficacy of the nomogram prediction.

### Gene Set Enrichment Analysis (GSEA) functional enrichment analysis

The function annotation was assessed using the R package “org.Hs.eg.db” and the collections of c2.cp.kegg.v7.5.1 and c5.go.v7.5.1 symbols. Gene sets with FDRs ≤ 0.05 were considered statistically significant. The “clusterProfiler” tool was used to analyze KEGG and Gene Ontology (GO) pathways, identifying enriched functions of histamine-related genes.

### Identification of the tumor immune infiltrating features of patients with HCC

The infiltrating immune cell ratio was measured using the Cell-type Identification. By Estimating Relative Subsets Of RNA Transcripts (CIBERSORT) method [21, 22] (https://cibersort.stanford.edu/). According to CIBERSORT, the proportion of 22 immune cells, including B-naive, B cell memory, plasma, CD8, CD4 naive, T cell follicular helper cells, CD4 memory resting, CD4 memory activation, regulatory T (Tregs), γδ, monocyte, NK, resting NK, macrophage M0, macrophage M1, macrophage M2, resting dendritic, activated dendritic, resting mast, activated mast, eosinophil, and neutrophil cells were assessed. The R package "ESTIMATE" was used to determine tumor purity, stromal, immunological, and estimate scores for each tumor sample [23, 24]. The immune infiltration in both groups was assessed using a single-sample Gene Set Enrichment Analysis (ssGSEA) technique based on 28 different immune cell types [25, 26].

### Chemotherapy sensitivity analysis

To evaluate the drug treatment response prediction capability of HRGPS, “pRRophetic” software package was employed to calculate the half-maximal inhibitory concentration (IC50) values of common chemotherapeutic drugs in the TCGA cohort. The differences in IC50 values between the high-risk and low-risk groups were compared using the Wilcoxon rank-sum test, and the results were visualized using "ggplot" tool in R.

### Identification of the appropriate population for immunotherapy

To evaluate the sensitivity of immunotherapy in both risk groups, we screened for expression levels of PD1, PD-L1, PD-L2, CTLA4, TIM-3, and TIGIT. Tumor immune dysfunction and exclusion (TIDE) algorithm [27] was utilized to predict the patient’s immunotherapy response. Higher TIDE scores were found to be associated with poor immunotherapy outcomes. In addition, we obtained an IMvigor210 dataset, which includes clinical data on atezolizumab for urothelial carcinoma. The T cell receptor (TCR) repertoire diversity was assessed using the richness and Shannon diversity index. The B cell receptor (BCR) isozymes were used to measure BCR abundance. Furthermore, it was observed that TCR and BCR richness could predict the intensity of immunotherapy response. The cancer testicular antigen (CTA) score reflects tumor immunogenicity and indirectly measures the response to immunotherapy. We compared the response to immunotherapy between high-risk and low-risk groups based on their TCR, BCR, and CTA scores.

### qRT-PCR validation of gene expression in the prognostic signature

To verify the prognostic signature gene expression, we collected tumors and corresponding paracancerous tissues from 10 patients diagnosed with HCC post-surgery. All patients with HCC signed an informed consent form before surgery. Quantitative real-time polymerase chain reaction (qRT-PCR) was used to confirm the expression of signature genes in HCC. Total RNA was isolated from the HCC tissues using Trizol, followed by reverse transcription to convert RNA into cDNAs. Subsequently, qRT-PCR was employed to evaluate the expression of signature genes in tumor and normal tissues of patients with HCC. The 2^−ΔΔCT^ method was used to calculate the signature genes’ relative expression values, normalized with β-actin. The sequences of primer pairs for the genes targeted are listed in Table S1.

## Statistical analysis

The Spearman correlation test assessed the nonlinear relationships between the two variables. Student's *t*-test was employed to compare the normally distributed data, while the Chi-square test compared categorical and pairwise subgroup traits. Wilcoxon test was applied to compare ordinal and non-normally distributed data between the subgroups. All statistical analyses were conducted using R 4.1.2 and GraphPad Prism 8. Survival analyses utilized KM and log-rank tests, with a significance threshold of *p* < 0.05.

## Results

### Development of a prognostic histamine-based risk score signature for HCC

**Fig. 1:**
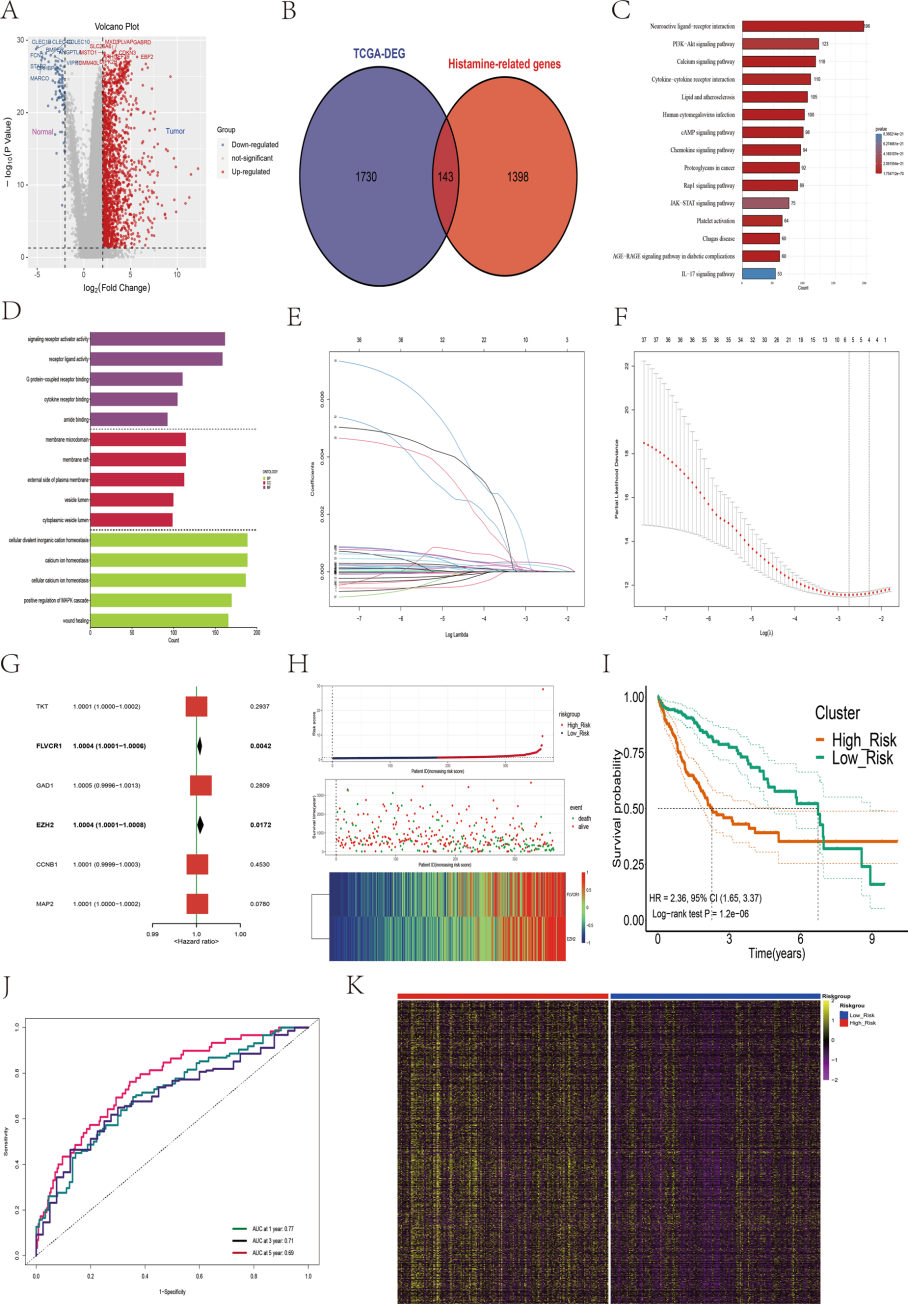
Construction of a prognostic signature based on the differentially expressed histamine-related genes. **A** Volcano map showing differentially expressed genes in HCC and paracancerous tissue samples. **B** Identification of differentially expressed histamine-related genes. **C** Results of KEGG functional enrichment analysis of 1541 histamine-related genes. **D** Results of GO functional enrichment analysis. **E** The coefficients of genes were calculated by multivariate Cox regression using LASSO. **F** The partial likelihood of deviance of genes. **G** Results of Cox multivariate analysis of six differentially expressed histamine-related genes. **H** The association of risk scores with survival status and gene expression in patients with HCC in the training set. **I** Kaplan–Meier curves were used to compare the overall survival of patients with HCC between the high- and low-risk groups in the training set. **J** ROC curves of the prognostic signature for predicting the risk of death at 1, 3, and 5 years in the training set. **K** The heatmap shows 1541 histamine-related differentially expressed genes in the high-risk and low-risk groups

The differential analysis of HCC and paracancerous tissue samples identified 1873 genes (Fig. 1A). Integration of histamine-related signaling pathways from the database revealed 1541 specific histamine-related genes. After intersecting these datasets, we identified 143 histamine-related DEGs for further analysis (Fig. 1B). After employing GO and KEGG analysis for functional pathway enrichment, the primary enriched pathways were the neuroactive ligand–receptor interaction, PI3K–Akt signaling pathway, and cytokine–cytokine receptor interaction (Fig. 1C). Histamine-related genes were predominantly enriched in the biological processes such as cellular divalent inorganic cation homeostasis, calcium ion homeostasis, and cellular calcium ion homeostasis signaling pathway (Fig. 1D). The Cox univariate regression and LASSO analyses (*p* < 0.05) revealed six histamine-related genes that have a significant impact on the prognosis of patients with HCC (Fig. 1E, F). *EZH2* and *FLVCR1* were determined to be independent risk factors affecting the prognosis of HCC through Cox multivariate regression analysis (*p* < 0.05; Fig. 1G). Risk scores were calculated for all the patients in TCGA training cohorts using a defined formula: RiskScore = 3.599e – 04 × *FLVCR1* expression + 5.473e – 04 × *EZH2* expression. The median risk score was used as the cutoff value to classify patients into high- and low-risk groups (Fig. 1H). Survival analyses revealed that patients in the high-risk group had worse overall survival (OS) than those in the low-risk group in the TCGA training cohort (*p* < 0.001; Fig. 1I). The area under the curve (AUC) for OS at respective time points was as follows: one year, 0.77; three years, 0.71; and five years, 0.69 (Fig. 1J). The heatmap demonstrated the overexpression of most histamine-related genes in the high-risk group (Fig. 1K), suggesting that high expression levels of these genes characterize this group.

### Relationship between HRGPS and hypoxic microenvironment

**Fig. 2:**
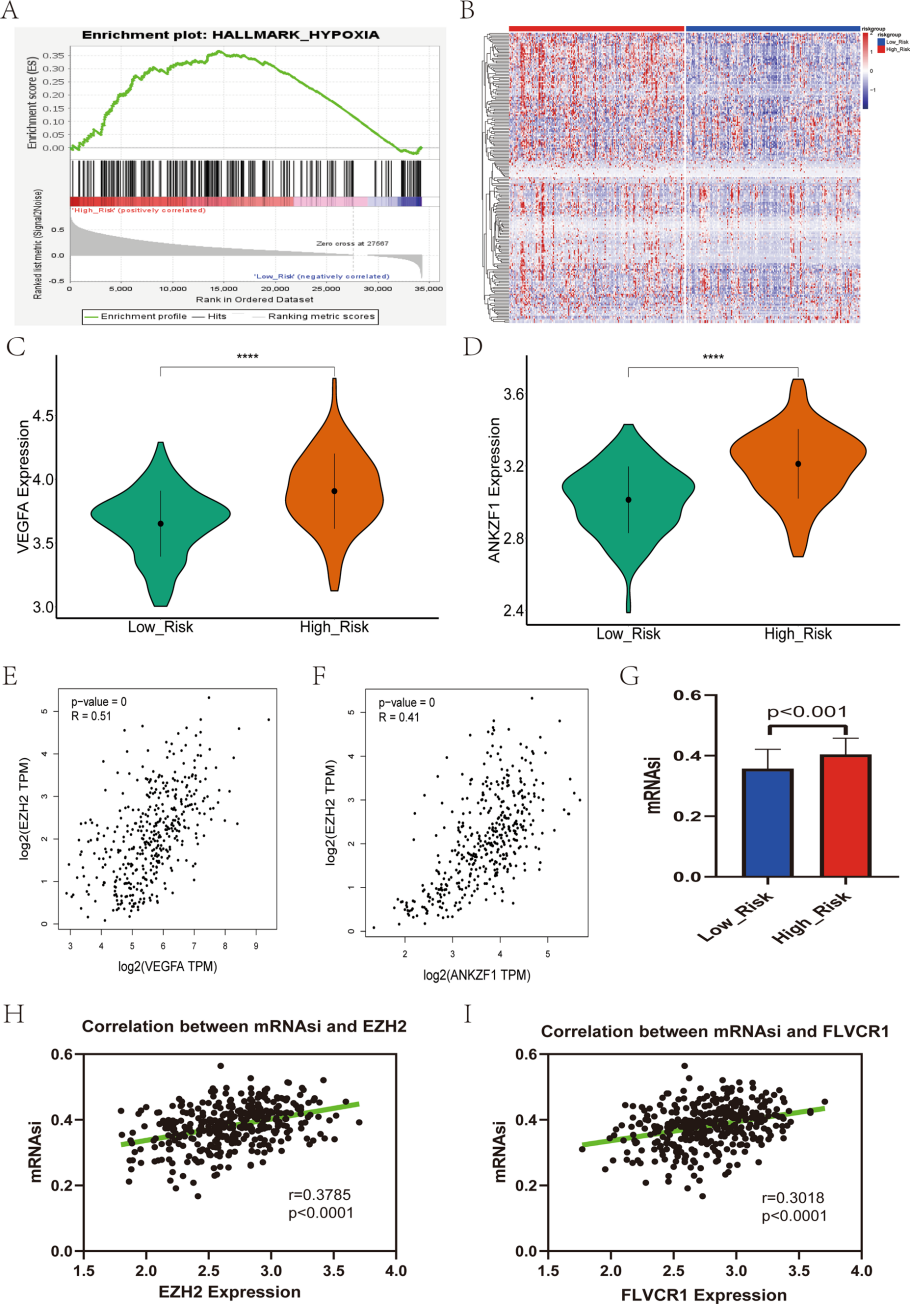
Relationship between the prognostic signature and hypoxic microenvironment. **A** The GSEA functional enrichment analysis results show that the hypoxia signaling pathway enriched the high-risk group. **B** Heatmap demonstrating that most hypoxia-related genes are overexpressed in high-risk groups. **C**–**D** The mRNA expression levels of two key genes (*VEGFA* and *ANKZF1*) in the hypoxic microenvironment were significantly higher in the high-risk group. **E** The mRNA levels of the *EZH2* were positively associated with those of the *VEGFA*. **F** The mRNA levels of the *EZH2* were positively associated with those of the *ANKZF1*. **G** The mRNAsi levels were significantly higher in the high-risk group than in the low-risk group. **H**–**I** A significant positive correlation was observed between the mRNA expression levels of histamine-related genes (*EZH2* and *FLVCR1*) and mRNAsi scores

The hypoxic microenvironment is critical in immune cell suppression and tumor cell invasion in the TME. GSEA enrichment analysis revealed that the high-risk group was predominantly enriched in the hypoxia signaling pathway (Fig. 2A). The majority of genes associated with hypoxia were found to be overexpressed in the high-risk group (Fig. 2B), including *VEGFA* and *ANKZF1* in the hypoxia pathway (Fig. 2C, D) [28, 29]. Correlation analysis indicated a strong relationship between mRNA expression levels of *VEGFA* and *ANKZF1* and histamine-related genes (Fig. 2E, F). These findings suggest that overexpression of histamine-related genes may result from the hypoxic microenvironment in tumor tissues.

Previous studies have suggested that the hypoxic tumor microenvironment could affect cancer stem-like cell phenotype [30, 31], prompting us to investigate the association between histamine-related genes and mRNAsi score. Notably, the high-risk group exhibited higher mRNAsi scores than the low-risk group (Fig. 2G). Correlation analysis also demonstrated a positive correlation between the mRNA expression levels of both *EZH2* and *FLVCR1* with mRNAsi scores (Fig. 2H, I). These findings suggest that in the hypoxic environment of HCC, overexpression of genes related to histamine may affect the phenotype of cancer stem cell-like cells.

### Association between HRGPS and tumor immunity

**Fig. 3:**
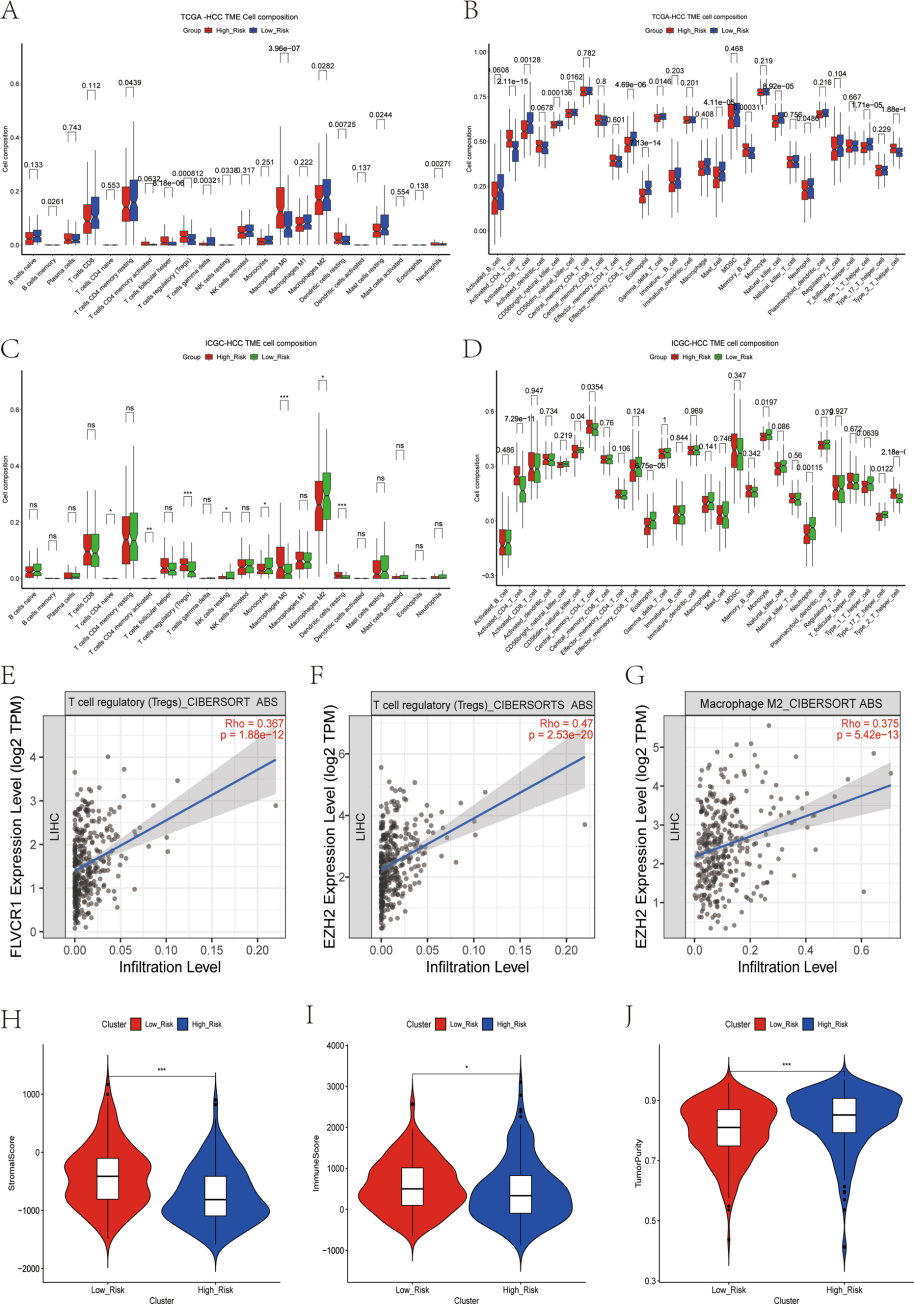
Relationship between the prognostic signature and immune microenvironment. **A** Differences in the proportion of infiltrating immune cells between the high- and low-risk groups in the TCGA database. **B** Differences in the proportion of 28 immune cells infiltrates in the TME between high- and low-risk groups in the TCGA database. **C** Differences in the proportion of infiltrating immune cells between the high- and low-risk groups in the ICGC database. **D** Differences in the proportion of 28 immune cells infiltrates in the TME between high- and low-risk groups in the ICGC database. **E** The mRNA expression level of *FLVCR1* was positively correlated with the proportion of Tregs infiltration. **F** The mRNA expression level of *EZH2* was positively correlated with the proportion of Tregs infiltration. **G** The mRNA expression level of *EZH2* was positively correlated with the proportion of macrophage infiltration. **H**–**J** Differences in StromalScore, ImmuneScore, and TumorPurity between the high- and low-risk groups

We investigated the correlation between histamine and the immune status of patients in the TCGA cohort and observed significant alterations in the immune cell populations. Interestingly, we noted a substantial infiltration of immunosuppressive cells in the TME, including Tregs, macrophages, dendritic cells, and neutrophils, particularly within the high-risk group, indicating pronounced immunosuppression (Fig. 3A). Similar outcomes were derived from ssGSEA enrichment analysis (Fig. 3B). The findings of the study of immune cell infiltration indicate that histamine-related genes could play a role in developing an immunosuppressive microenvironment by recruiting immunosuppressive cells and helping tumor cells avoid immune surveillance. To further validate this result, we applied the same methodology to calculate immune cell infiltration abundance in a validation set comprising 220 patients with HCC (Fig. 3C, D). The results were consistent with those obtained in the training set. Correlation analysis revealed a positive association between *EZH2* and *FLVCR1* expression levels with infiltrating Treg cells and macrophages (Fig. 3E–G). Utilizing the ESTIMATE algorithm, it was determined that individuals within the high-risk group exhibited lower StromalScore and ImmuneScore but higher TumorPurity (Fig. 3H–J).

### Correlations between HRGPS and clinical characteristics

**Fig. 4:**
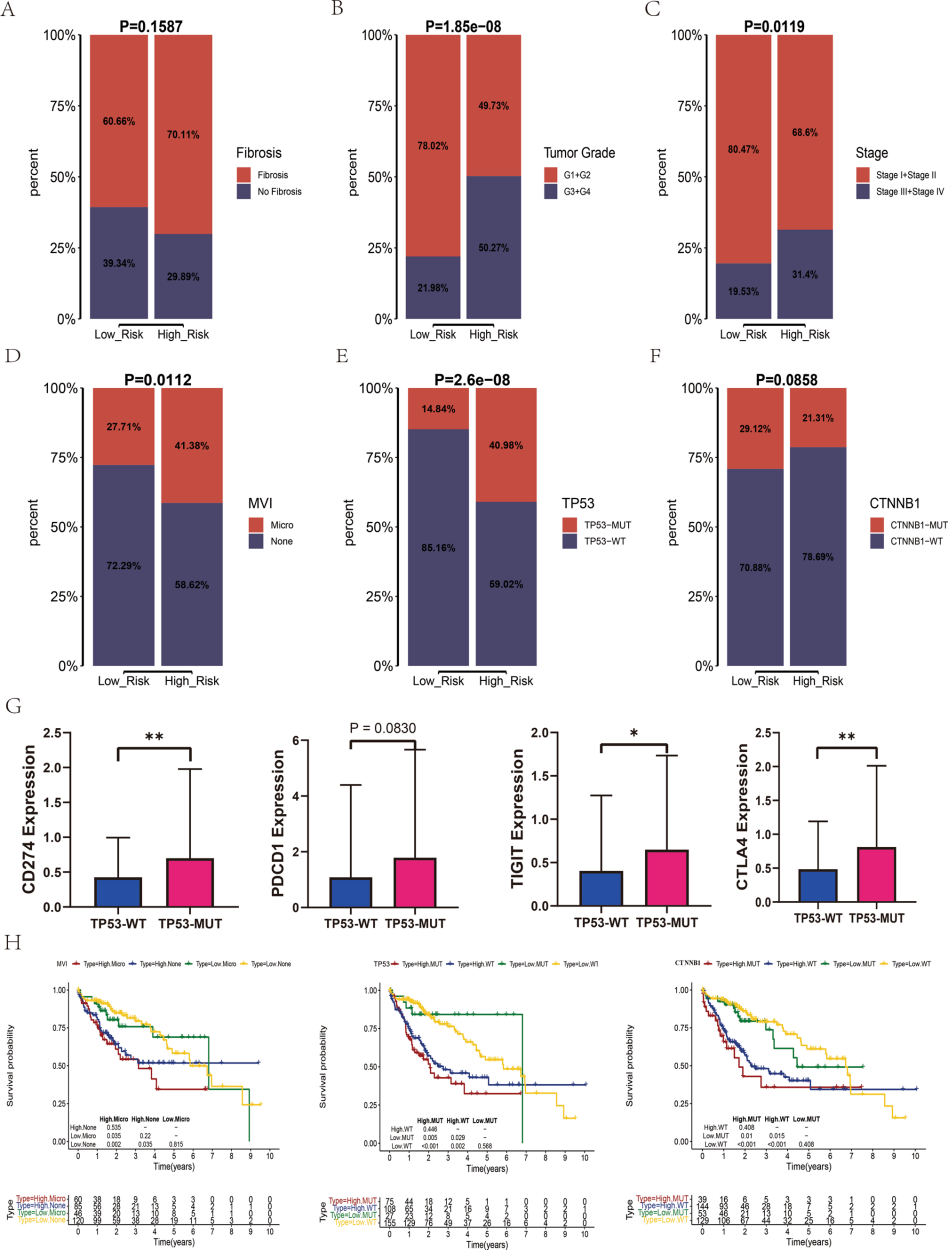
The relationship between risk scores and clinical characteristics. **A**–**C** The relationship between risk score and clinical characteristics such as background of liver fibrosis, tumor stage, and tumor grade. **D**–**F** The relationship between risk score and MVI, *TP53*, and *CTNNB1* mutations. **G** The relationship between immune checkpoint gene expression and *TP53* mutations. **H** Impact of risk scores and MVI, *TP53,* and *CTNNB1* mutations on the prognosis of patients with HCC

We explored the association between HRGPS and clinical characteristics. Notably, patients with liver fibrosis, more advanced tumor stage (Stage III + IV), and higher tumor grade (G3 + G4) were primarily distributed in the high-risk group (Fig. 4A–C). In addition, microvascular invasion (MVI) is an important risk factor for early recurrence and metastasis of HCC after surgery. We found many patients with MVI-positive HCC in the high-risk group (Fig. 4D), suggesting that the risk score can predict early recurrence and metastasis of HCC. The frequency of *TP53* somatic mutations in patients with HCC in the high-risk group was 40.98%, significantly higher than the 14.48% in the low-risk group (Fig. 4E). Conversely, mutations in *CTNNB*1 showed the opposite pattern (Fig. 4F). It was demonstrated that there was a strong correlation between the *TP53* mutation and the TME of HCC, wherein FOXP3 + Tregs were more prevalent, and CD8 + T cells were less invasive, leading to a downregulated immune response. Our study showed patients with *TP53* mutations overexpressed immune checkpoint molecules (such as PD1, PD-L1, TIGIT, and CTLA4) (Fig. 4G). These results suggest that *TP53* mutations enhanced the expression of the tumor cell surface molecule PD1 and immune cell surface molecules PD-L1, TIGIT, and CTLA4. This indicates that patients with HCC in the high-risk group are more likely to be responsive to immunotherapy. Survival analysis showed that patients with HCC with MVI-negative, *TP53* wild-type, and *CTNNB1* wild-type in the low-risk group had a better prognosis (Fig. 4H).

**Fig. 5:**
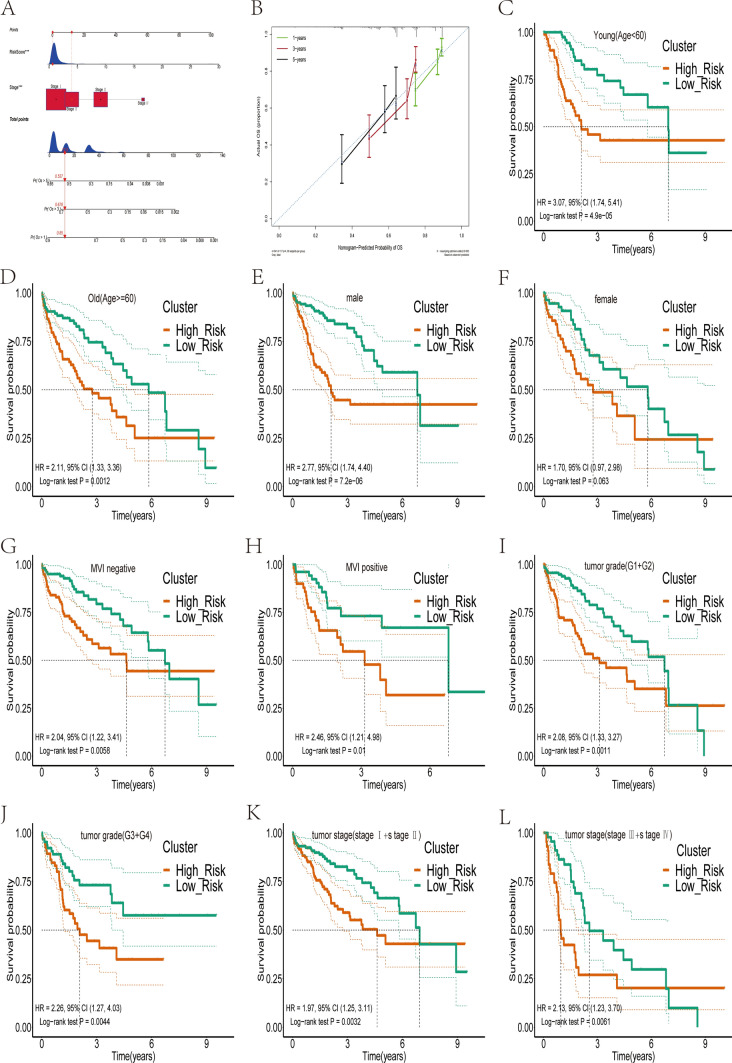
Construction of nomogram and internal validation of prognostic signature. **A** A nomogram was constructed based on the tumor stage and risk score. **B** Calibration curves showing strong predictive power of nomogram for 1-, 3-, and 5-year survival in patients with HCC in TCGA database. **C**–**L** The validity of prognostic signature was validated in different clinical subgroups, including young (**C**), old (**D**), male (**E**), female (**F**), MVI-negative (**G**), MVI-positive (**H**), low tumor grade (**I**), high tumor grade (**J**), early stage (**K**), and advanced stage(**L**)

Both univariate and multivariate Cox analyses demonstrated that the risk score independently influenced the prognosis of patients with HCC (Table 1). Furthermore, the ROC curves indicated that the risk score had superior predictive power compared to other clinical characteristics (Figure S2A). Subsequently, we constructed a nomogram based on the risk score and tumor stage, which could effectively predict the prognosis of patients with HCC (Fig. 5A). Calibration and ROC curves showed that the nomogram accurately predicted patients with HCC survival at 1, 3, and 5 years (Fig. 5B, Figure S2B). To further assess the robustness of the risk scores in predicting the prognosis of patients with HCC, we first stratified the patients according to age (Fig. 5C, D), gender (Fig. 5E, F), MVI (Fig. 5G, H), tumor grade (Fig. 5I, J), and tumor stage (Fig. 5K, L). After stratification, OS was significantly lower in all high-risk groups except for the female subgroup.

**Table 1:** Results of univariate and multivariate Cox analyses of clinical characteristics and risk scores of HCC patients in the training set

Characteristics	Univariate analysis		Multivariate analysis	
	HR (95% CI)	*p*	HR (95% CI)	*p*
Age	1.011 (0.997–1.026)	0.125		
Gender				
Female	Reference			
Male	0.792 (0.545–1.150)	0.221		
Grade stage				
Grade 1 + 2	Reference			
Grade 3 + 4	1.186 (0.819–1.716)	0.366		
M stage				
M0	Reference			
MX	1.392 (0.915–2.117)	0.122		
N stage				
N0	Reference			
NX	1.228 (0.845–1.961)	0.239		
Tumor Stage				
Stage I + II	Reference			
Stage III + IV	2.409 (1.664–3.487)	< 0.001	2.241 (1.537–3.270)	< 0.001
RiskScore	1.140 (1.087–1.196)	< 0.001	1.116 (1.061–1.173)	< 0.001

### Validation of the prognostic signature

To assess the predictive robustness of HRGPS, we computed the risk scores for two independent cohorts using the same formula as the training set. Subsequently, patients were stratified into low- and high-risk groups according to the median risk score. According to the findings, patients in the low-risk group had a better OS than those in the high-risk group (Figure S3A–S3B). However, HRGPS exhibited high AUC values in predicting the probabilities of 1-, 3-, and 5-year survival (Figure S3C–S3D), suggesting its excellent performance in predicting the prognosis of patients with HCC. In the two external independent datasets, expression levels of *EZH2* and *FLVCR1* were found to increase concomitantly with higher risk scores (Figure S3E–S3F).

### Prediction of immunotherapy efficacy by the HRGPS

**Fig. 6:**
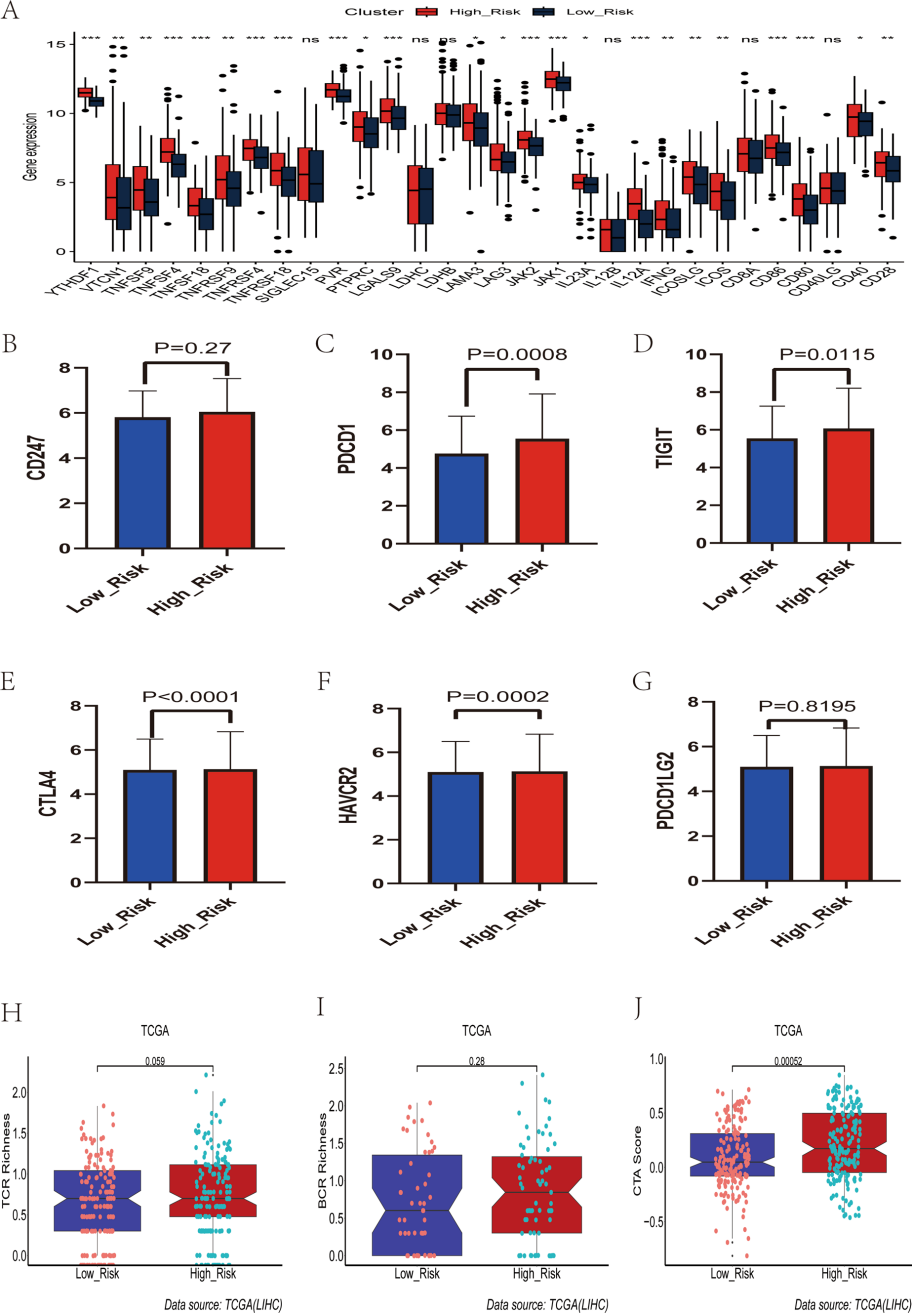
Prediction of immunotherapy response by prognostic signature. **A** Relationship between the mRNA expression levels of immune checkpoint-related genes and prognostic signature. **B**–**G** Differences in mRNA expression levels of immune checkpoint-related genes commonly used in HCC immunotherapy in high- and low-risk groups. **H**–**J** Differences in TCR richness, BCR richness, and CTA scores between the high- and low-risk groups

**Fig. 7:**
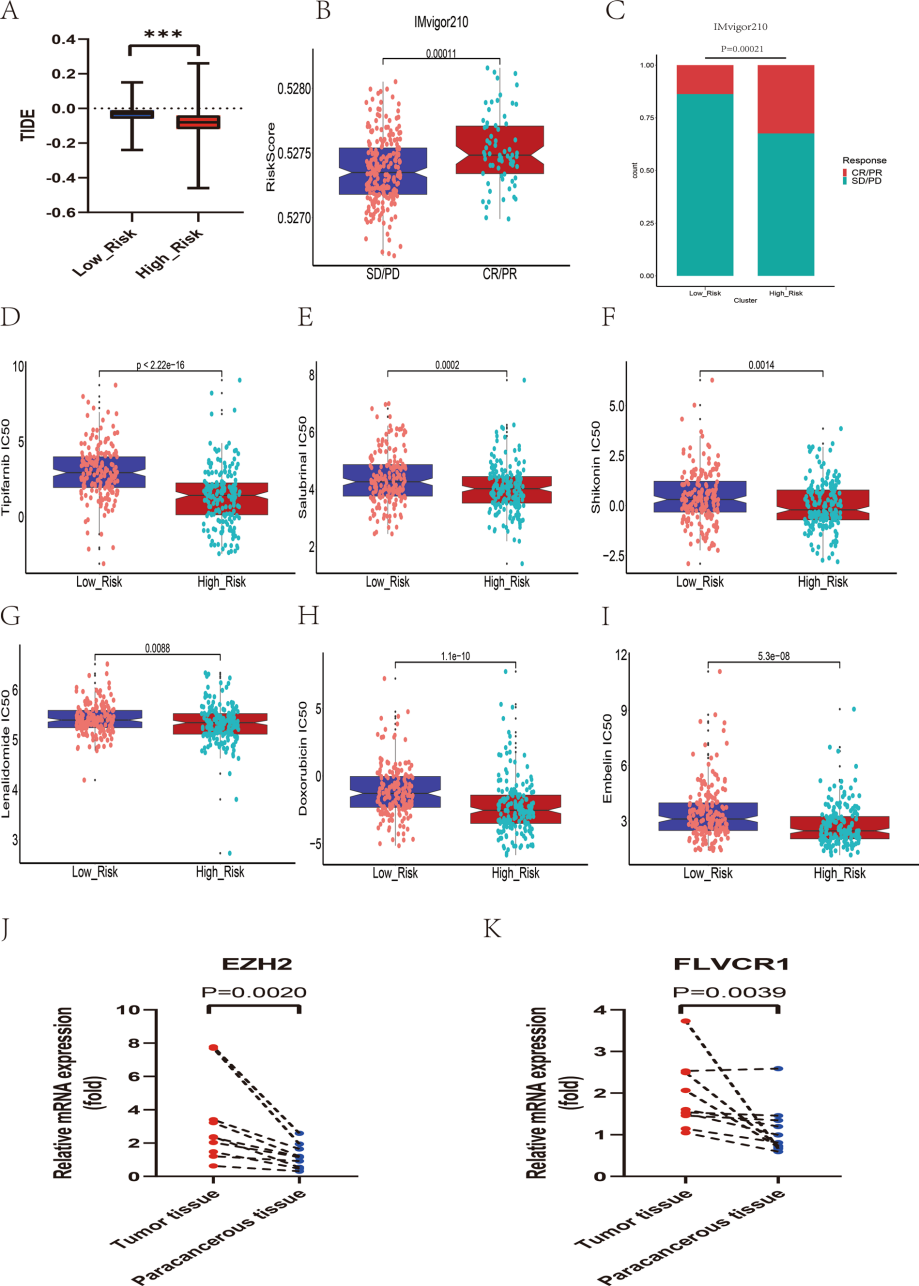
Prediction of immunotherapy response and chemotherapeutic drugs by prognostic signature. **A** Differences in the distribution of TIDE scores between the high- and low-risk groups. **B** In the IMvigor210 dataset, patients who responded to immunotherapy had significantly higher risk scores than the low-risk group. **C** A higher proportion of patients in the high-risk group responded to immunotherapy compared to the low-risk group in the IMvigor210 dataset. **D**–**I** Higher risk scores were associated with lower IC50 values for anticancer drugs such as Embelin, Salubrinal, Shikonin, Lenalidomide, Doxorubicin, and Tipifarnib. **J** EZH2 was overexpressed in HCC tissues in clinical samples compared to paraneoplastic tissues. **K** FLVCR1 was overexpressed in HCC tissues in clinical samples compared to paraneoplastic tissues

Based on the aforementioned study of immune cells, we further investigated whether histamine regulates the immune checkpoints, which are important regulators of TME, and their connection to HRGPS-based risk score. The expression levels of immune checkpoint-related genes play a crucial role in HCC immunotherapy, and our findings indicate that *YTHDF1*, *CD86*, and *CD80* were significantly overexpressed in the high-risk group (Fig. 6A). Furthermore, all six immune checkpoint genes showed higher expression in the high-risk group compared to the low-risk group, as observed from our initial comparison of *PDCD1*, *TIGIT*, *TIM-3*, and *CTLA4* expression patterns (Fig. 6B–G). These findings indicate that immunotherapy shows promise in reducing the immunosuppressive conditions observed in the high-risk group. In addition, rearrangement analysis of TCR and BCR was used to stratify and monitor the patients undergoing immunotherapy by identifying MHC-presented antigens. The cancer testicular antigen (CTA) score is an indirect measure of the tumor immunogenicity and thus indicates a potential response to immunotherapy. We found that the high-risk group exhibited higher TCR, BCR, and CTA scores than the low-risk group (Fig. 6H–J), suggesting a possible positive response to immunotherapy. Furthermore, TIDE was utilized to predict responses to immunotherapy. Interestingly, the high-risk individuals displayed lower TIDE scores (Fig. 7A). We also calculated risk scores for IMvigor210 patients. The complete/partial response (CR/PR) group had higher risk scores than the stable/progressive disease (SD/PD) group (Fig. 7B), and greater high-risk patients responded positively to immunotherapy (Fig. 7C).

### Analysis of response to chemotherapy

The calculated IC50 varied significantly between the two risk populations. The fact that Embelin, Salubrinal, Shikonin, Lenalidomide, Doxorubicin, and Tipifarnib IC50 values were lower in the high-risk group than in the low-risk group (Fig. 7D–I) suggests that patients with high-risk scores may be more sensitive to these chemotherapeutic agents.

### Verification of signature genes

We assessed the *EZH2* and *FLVCR1* mRNA expression levels in 10 pairs of HCC and paraneoplastic tissue samples. The results showed that the expression levels of *EZH2* and *FLVCR1* were higher in HCC samples compared to paracancerous tissues (*p* < 0.01) (Fig. 7J, K), suggesting that these genes may be involved in the progression of HCC.

## Discussion

HCC is a highly heterogeneous malignancy with a complex etiology. High morbidity and mortality of HCC have prompted worldwide concern despite advances in treatment. Inadequate prospective and verified research has resulted in a lack of clinically applicable biomarkers for HCC management [32]. So, it is critical to find risk classification tools and prognostic factors that are reliable and complementing. As the primary type of liver cancer, HCC is thought to be mainly associated with injury and long-term inflammation, with immune cell infiltration into the liver [33]. Histamine is a biologically active substance that potentiates the inflammatory and immune responses of the body and acts as neurotransmitter [34–36]. Potential cancer therapeutic agents include anti-inflammatory medications (AHs), as chronic inflammation often contributes to the development of cancer. Fritz et al. [37] demonstrated an association between the use of specific AHs and improved breast cancer survival. Other studies have reported similar outcomes in non-localized malignancies, non-small cell lung cancer, and ovarian cancer, and some commonly used antihistamines may also have antitumor effects [38]. These results suggest that histamine is actively involved in cancer cell proliferation, migration, and invasion. However, the significance of histamine in HCC etiology and its impact on immunotherapy is not fully understood.

We analyzed the transcriptomic data of patients with HCC and constructed a prognostic signature that can effectively predict the prognosis of HCC. To investigate the molecular mechanisms of HRGPS, we first performed a GSEA functional enrichment analysis on the high- and low-risk groups. The results indicated that the high-risk group was primarily associated with hypoxia signaling pathways. Previous research suggests that in the hypoxic microenvironment, a characteristic of tumors, tumor cells can enhance their ability to adapt by modifying their metabolism, suppressing the immune system's antitumor responses, and increasing the probability of invasion, metastasis, and activation of genes that confer resistance to drugs [39, 40]. We discovered an intriguing correlation between the expression levels of histamine-related and hypoxia-related genes. The hypoxic state in the TME can shape the phenotype of cancer stem cell-like cells [30]. Our study found that the high-risk group had significantly higher mRNAsi scores than the low-risk group. Additionally, we observed a robust positive correlation between the expression levels of histamine-related genes and mRNAsi scores. Our research findings and previous studies suggest that a hypoxic environment is responsible for the increased expression of genes related to histamine. Tumor cells can enhance their glycolysis level by adapting to the hypoxic environment or being influenced by histamine family members, which ultimately promotes tumor progression.

Histamine is an essential inflammatory factor in the human body, so we hypothesized that there is a close link between the expression of histamine-related genes and immune cell infiltration in TME. Notably, the percentage of immune cells with immunosuppressive functions, including Treg cells, macrophages, dendritic cells, and neutrophils, was significantly higher in the high-risk group than in the low-risk group. This was validated in an external independent dataset. According to these findings, histamine may encourage the migration and invasion of tumor cells by luring immune-suppressive cells to create an immunosuppressive microenvironment. This may partially explain the poor prognosis of patients in the high-risk group. However, the detailed molecular mechanisms need to be confirmed by further investigations.

Immunotherapy is a treatment for advanced liver cancer involving immune checkpoint-related gene expression and immune cell infiltration. In the study, mRNA expression of immune checkpoint-related genes was significantly higher in the high-risk group than in the low-risk group. Immunotherapy may be beneficial for high-risk patients. The TIDE score was used to assess the response to immunotherapy in patients with tumors. Lower TIDE scores predicted a more robust immunotherapy response. Immunotherapy is more likely to be effective for the high-risk group identified by the prognostic signature, as they have a lower TIDE score. To confirm our findings, we collected data on immunotherapy in patients with HCC and found that high-risk patients had a more significant response to immunotherapy. This supports the idea that immunotherapy is more appropriate for people at high risk for HCC. Thus, by accurately identifying immunotherapy candidates, our HRGPS will improve response rates to immunotherapy.

Our HRGPS better predicted the prognosis and treatment response of patients with HCC. However, this study has some limitations. First, the study used public datasets and some experimental validation, but more in vitro and in vivo experiments are needed to corroborate our conclusions. Second, the mechanism linking hypoxia and histamine production must be confirmed and studied. Third, more clinical tissue samples are required to study the effectiveness and robustness of HRGPS.

## Conclusion

By analyzing the transcriptomic data, we put forward HRGPS to effectively predict the prognosis and immunotherapy response in HCC. The study demonstrated a strong correlation between genes related to histamine and hypoxia, and the underlying mechanism was elucidated using our findings and previous research. Our study highlights the vital role of histamine and provides new insights to explore the mechanism of histamine-related genes involved in HCC.

## Supplementary Information

Below is the link to the electronic supplementary material.

Supplementary file1 (DOCX 8845 kb)

Supplementary file2 (XLSX 8 kb)

## Data Availability

Data availability Gene expression profiles, clinical information, and mutation data of HCC in this study are available from the public database (TCGA, https://portal.gdc.cancer.gov/). The immunotherapy dataset was downloaded from IMvigor210 (http://research-pub.gene.com/IMvigor210CoreBiologies/packageVersions/). GSE14520 was downloaded from the GEO database. The ICGC-LIHC data in the validation set were downloaded from the International Cancer Genome Consortium (ICGC, https://dcc.icgc. org/).
