# Supplementary material for: Histamine-related genes participate in the establishment of an immunosuppressive microenvironment and impact the immunotherapy response in hepatocellular carcinoma
Source: Clin Exp Med. 2024 Jun 17;24(1):129. doi: 10.1007/s10238-024-01399-9 (PMC11182831; doi:10.1007/s10238-024-01399-9)
Supplement: Supplementary file 1 — Supplementary file1 (DOCX 8845 kb) [file 10238_2024_1399_MOESM1_ESM.docx]

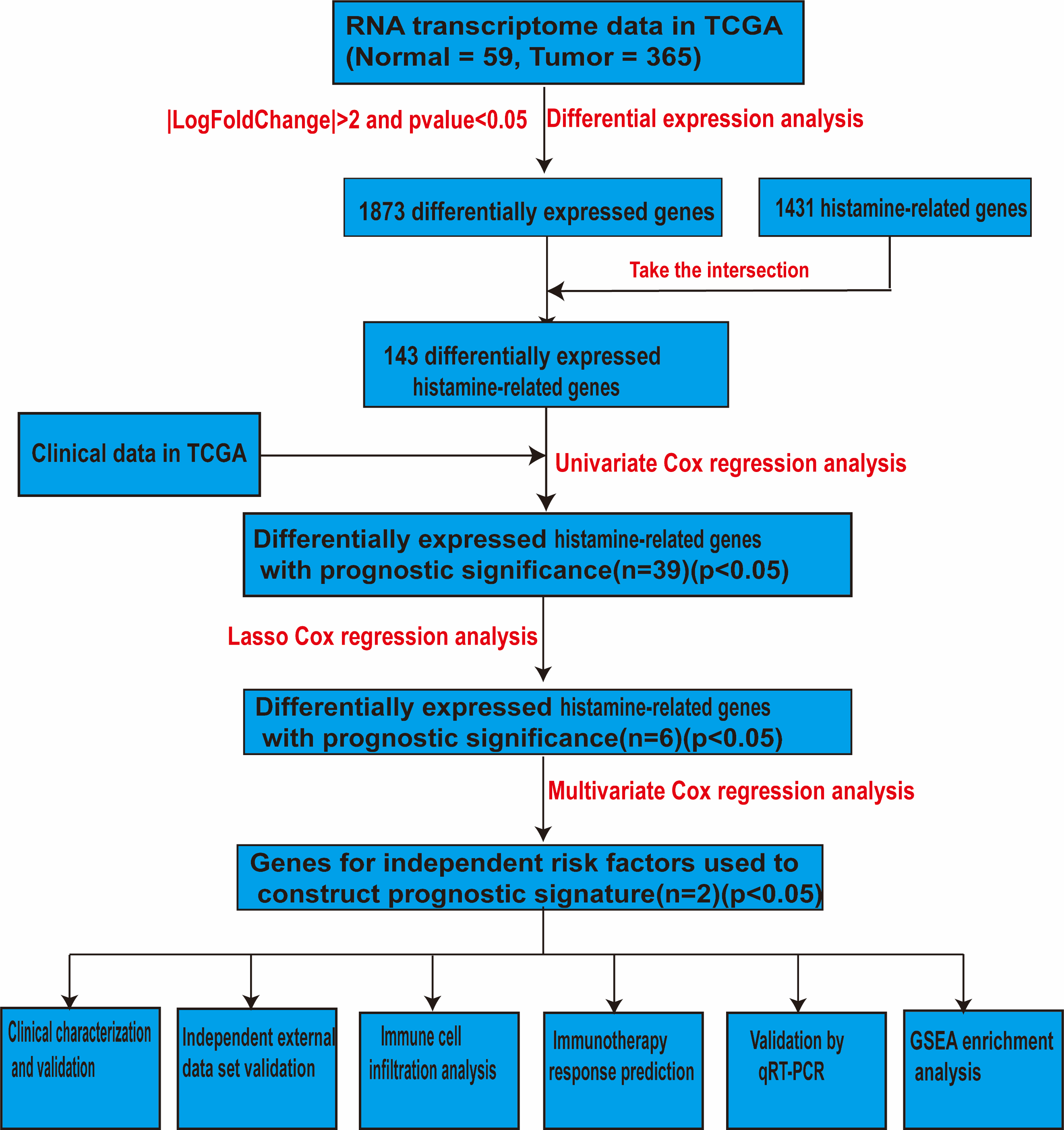


**Figure S1** Flowchart of the data analysis procedures in this study.

.

s
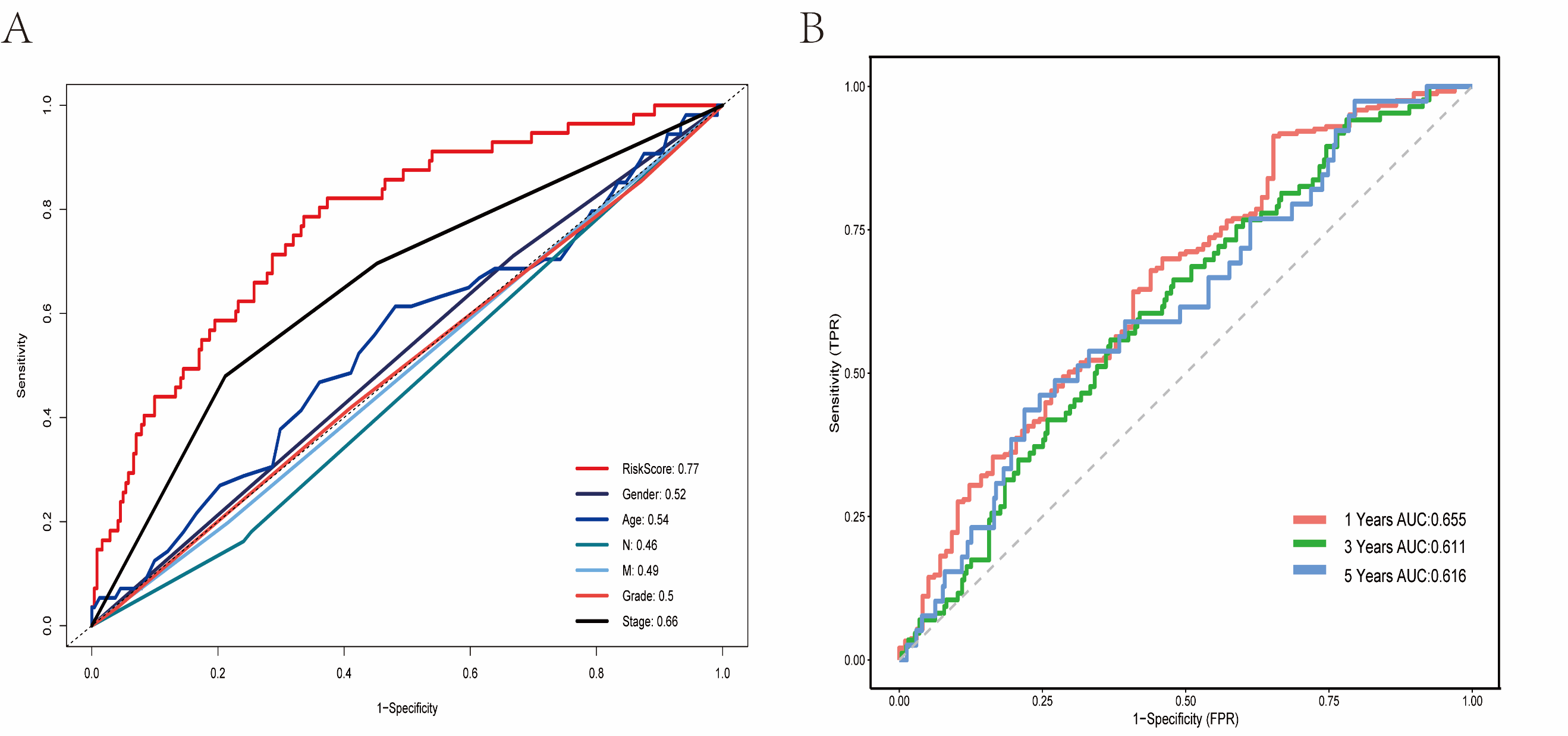


**Figure S2** Validation of prognostic signature for predicting the efficacy of prognosis in HCC patients. (A) ROC curves confirm that risk scores are more predictive of prognosis in HCC patients compared to other clinical characteristics. (B) ROC curves showing the results of nomogram for predicting 1-, 3-, and 5-year survival in HCC patients.


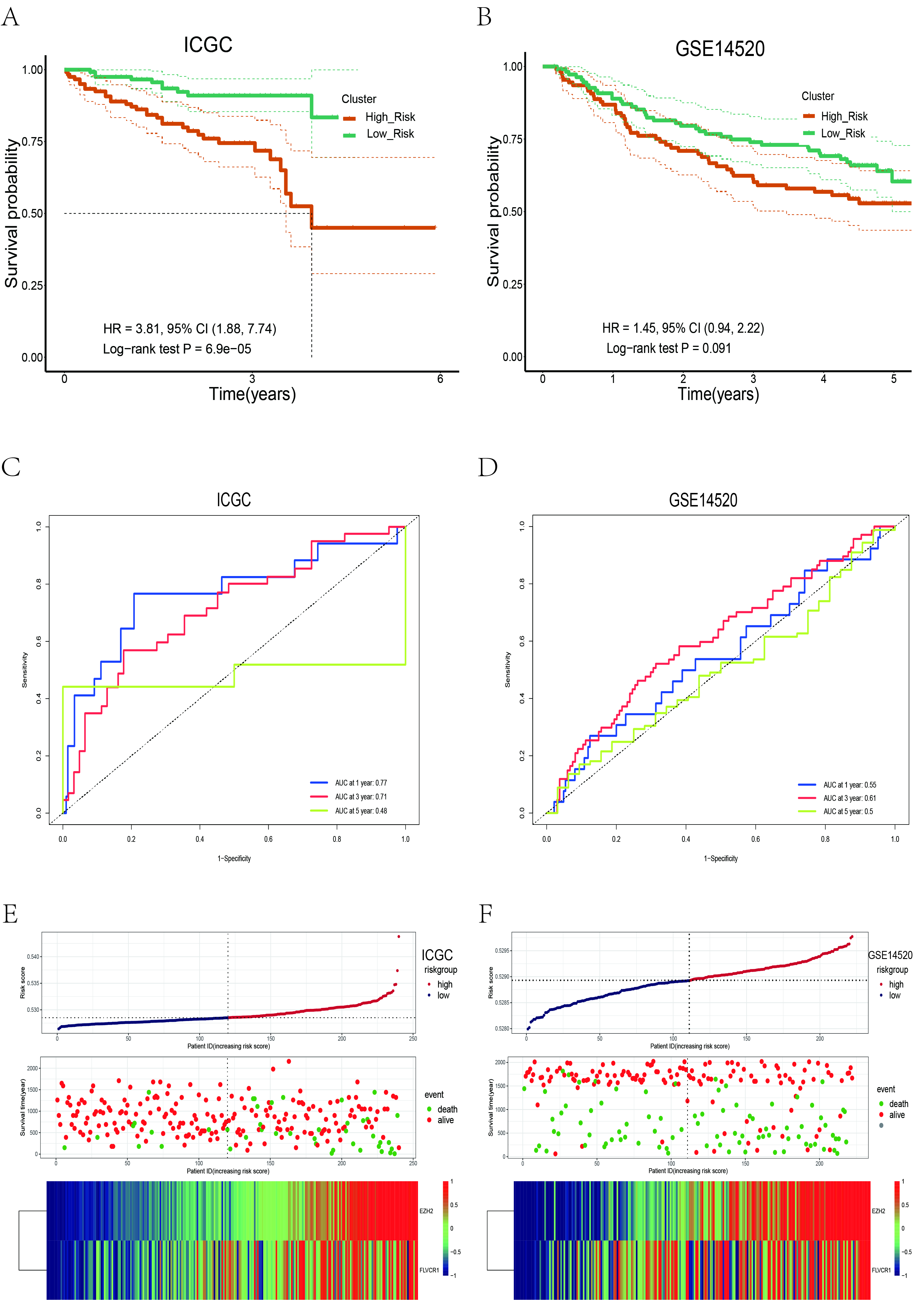


**Figure S3** External independent dataset for validation of prognostic signature. (A) (A) In the ICGC dataset, the overall survival time of patients in the high-risk group was significantly better than that of patients in the low-risk group. (B) In the GSE14520 dataset, the overall survival time of patients in the high-risk group was significantly better than that of patients in the low-risk group.(C-D) ROC curves confirmed that the prognostic model has good predictive efficacy for overall survival time at 1, 3 and 5 years in HCC patients in the ICGC and GSE14520 databases, respectively. (E-F) The expression levels of histamine-related genes (EZH2 and FLVCR1) increased with increasing risk scores in the ICGC and GSE14520 databases, respectively.
